# *n*-Hexane Insoluble Fraction of *Plantago lanceolata* Exerts Anti-Inflammatory Activity in Mice by Inhibiting Cyclooxygenase-2 and Reducing Chemokines Levels

**DOI:** 10.3390/scipharm85010012

**Published:** 2017-03-13

**Authors:** Nanang Fakhrudin, Eny Dwi Astuti, Rini Sulistyawati, Djoko Santosa, Ratna Susandarini, Arief Nurrochmad, Subagus Wahyuono

**Affiliations:** 1 Faculty of Pharmacy, Universitas Gadjah Mada, Sekip Utara, Yogyakarta 55281, Indonesia; aenydwi10@gmail.com (E.D.A.); sulistyawati.rini@yahoo.co.id (R.S.); djoko5346@ugm.ac.id (D.S.); ariefnr@ugm.ac.id (A.N.); subagusw@yahoo.com (S.W.); 2 Center for Natural Antiinfective Research, Faculty of Pharmacy, Universitas Gadjah Mada, Sekip Utara, Yogyakarta 55281, Indonesia; 3 Faculty of Biology, Universitas Gadjah Mada, Sekip Utara, Yogyakarta 55281, Indonesia; rsusandarini@gmail.com; 4 Akademi Analis Farmasi Al Islam Yogyakarta, Gedongkiwo, Mantrijeron, Yogyakarta 55142, Indonesia

**Keywords:** *Artocarpus altilis*, anti-inflammation, chemokines

## Abstract

Inflammation is involved in the progression of many disorders, such as tumors, arthritis, gastritis, and atherosclerosis. Thus, the development of new agents targeting inflammation is still challenging. Medicinal plants have been used traditionally to treat various diseases including inflammation. A previous study has indicated that dichloromethane extract of *P. lanceolata* leaves exerts anti-inflammatory activity in an in vitro model. Here, we examined the in vivo anti-inflammatory activities of a *n*-hexane insoluble fraction of *P. lanceolata* leaves dichloromethane extract (HIFPL). We first evaluated its potency to reduce paw edema induced by carrageenan, and the expression of the proinflammatory enzyme, cyclooxygenase (COX)-2, in mice. The efficacy of HIFPL to inhibit COX-2 was also evaluated in an in vitro enzymatic assay. We further studied the effect of HIFPL on leukocytes migration in mice induced by thioglycollate. The level of chemokines facilitating the migration of leukocytes was also measured. We found that HIFPL (40, 80, 160 mg/kg) demonstrated anti-inflammatory activities in mice. The HIFPL reduced the volume of paw edema and COX-2 expression. However, HIFPL acts as an unselective COX-2 inhibitor as it inhibited COX-1 with a slightly higher potency. Interestingly, HIFPL strongly inhibited leukocyte migration by reducing the level of chemokines, Interleukine-8 (IL-8) and Monocyte chemoattractant protein-1 (MCP-1).

## 1. Introduction

Inflammation is a biological process disrupting tissue homeostasis that involves the accumulation and recruitment of blood-derived products (leukocytes, fluid, plasma protein) into perturbed tissue and induces vasodilatation, vascular permeability and augmented blood flow [1]. Inflammation is also related with various pathological conditions such as arthritis, cancer, sepsis, metabolic, neurodegenerative and cardiovascular diseases [2,3]. To date, corticosteroids and non-steroidal anti-inflammatory drugs (NSAID) are available in clinical practices. These drugs remain the common choice to cure inflammatory diseases. However, severe side effects and lack of potency in reducing a specific inflammatory symptom restricted the usage of these drugs. Furthermore, gastrointestinal tract-related toxicity often occurs because of high-dose NSAID consumption, whereas long-term medication using corticosteroids anti-inflammatory drugs could lead to osteoporosis, weight gain, and immunosuppressive effects [4]. Many efforts have been made to identify potential targets of anti-inflammation and to find promising anti-inflammatory agents from various sources [5,6].

One of the most important and well-established therapeutic targets is cyclooxygenase (COX). This enzyme is responsible for the biosynthesis of the inflammatory mediator, prostanoids from arachidonic acid, and displays a role for therapeutic intervention in pain and inflammation [7,8]. COX-1 is constitutively expressed and facilitates the production of prostaglandins and thromboxane A2. It is also involved in the regulation of vascular, renal, gastrointestinal and various physiological functions. COX-2 is an inducible enzyme expressed during the inflammatory processes [9]. This enzyme facilitates prostaglandins production and mediates fever, pain and inflammatory processes [7,9,10]. Although COX-2 is one of the best-characterized therapeutic targets [10,11], COX-2 inhibition is often accompanied with undesired side effects, such as renal and gastrointestinal toxicities [11,12]. Selective COX-2 inhibitors “Coxibs” derivatives, for instance valdecoxib and rofecoxib, have been developed, but they have been withdrawn due to serious skin reactions and increased risks of heart attack and stroke, respectively [13,14]. Thus, the exploration and development of alternative anti-inflammatory agents from different sources is still a challenging area of research.

Medicinal plants provide abundant biodiversity of natural compounds with a huge structural variety and represent a potential source for drug discovery and development. One of the promising plants with anti-inflammatory activity is *Plantago lanceolata* L. (Plantaginaceae)*.* The leaves of *P. lanceolata* have been traditionally used to cure inflammatory disorders, such as inflammation of skin, oral, pharyngeal mucosa, respiratory tract, mouth and the throat [15]. In a previous study [16], the corresponding author focussed on the ethnopharmacological in vitro studies on folk medicine for anti-inflammation by performing a screening for anti-inflammatory activity from a collection of medicinal plants traditionally used to cure inflammatory diseases. The study found that the dichloromethane extract of *P. lanceolata* leaves inhibited nuclear factor-kappa B (NF-κB) activation induced by tumour necrosis factor-alpha (TNF-α). Here, we further investigated the anti-inflammatory effect of dichloromethane extracts of *P. lanceolata* leaves on mice. Chlorophylls and other non-polar inert constituents were separated by *n*-hexane partition to give a *n*-hexane insoluble fraction of *P. lanceolata* leaves extract (HIFPL) containing the more polar constituents of dichloromethane extract. 

## 2. Materials and Methods

### 2.1. Plant Material

*Plantago lanceolata* leaves were collected from Tawangmangu, Karanganyar, Jawa Tengah, Indonesia. Plant species determination was done by the botanist (Djoko Santosa) and the voucher specimen (number NF-01-02-TM-14) was deposited at the Dept. of Pharmaceutical Biology, Faculty of Pharmacy, Universitas Gadjah Mada, Indonesia. The fresh leaves were dried in the oven set at 50 °C for 48 h. The dried leaves were ground and stored at 4 °C until used for extraction.

### 2.2. Extraction and Fractionation

The powdered plant material (1000 g) was macerated with dichloromethane (5 L) at room temperature for 24 h. After filtration, the residue was remacerated with dichloromethane in a similar procedure. The filtrates were combined and evaporated at reduced pressure using a rotary evaporator for drying. The dried extract (13.8 g) was then dissolved in 69 mL *n*-hexane to produce *n*-hexane soluble and insoluble (HIFPL) fractions. The fractionation process was repeated five times until colorless *n*-hexane soluble fraction was obtained. This method yielded 8.6 g and 4.9 g of *n*-hexane soluble and insoluble fractions, respectively. The HIFPL was subjected to anti-inflammatory activity assays.

### 2.3. Animal Experiments

#### 2.3.1. Animal

The animals used for the anti-inflammatory study were mice (*Mus musculus*, BALB/c strain, 20–30 g of weight). The mice were maintained in an animal house with a controlled environment. They were fed with a standard pellet diet, and water was given ad libitum. Before the experiment, the mice were acclimatized in the animal house for a week, randomized and further divided into five groups. The experiment procedures were approved by Institutional Animal Ethic Committee at the Integrated Research and Testing Laboratory, Universitas Gadjah Mada (number 191/KEC-LPPT/IX/2014).

#### 2.3.2. Carrageenan-Induced Paw Edema in Mice

A carrageenan-induced mice paw edema assay was done as previously described [17] to study the inflammatory potency of HIFPL. The right hind paw of the mice was injected (sub plantar) with 0.2 mL carrageenan 1% (freshly prepared). The mice were divided randomly into five different groups (five mice in each group). Thirty minutes prior to the injection of carrageenan, the HIFPL-treated groups received orally a single dose of 0.1 mL HIFPL (40, 80 or 160 mg/kg) whereas the solvent- and indomethacin-treated groups were orally given 0.1 mL solvent (DMSO) and indomethacin (5 mg/kg), respectively. After carrageenan injection, the volume of the edema was measured every 30 min for 6 h using a plethysmometer instrument. The anti-inflammatory activity was determined with the trapezoidal method [18] by plotting the time of induction (from 0 to 6 h) versus paw edema to obtain the area under the curve (AUC). The anti-inflammatory activity of HIFPL was based on the percentage of the AUC compared to the solvent-treated group.

#### 2.3.3. Immunohistochemistry

After determination of the volume of the edema, the mice were sacrificed and the hind paw (soft plantar region sections) were cut and fixed [19]. The paraffin-embedded sections were deparaffinized, dehydrated, washed in a phosphate buffered saline solution (PBS) and then incubated in a peroxidase blocking solution followed with mice primary antibody (anti COX-2; 1:250) at 4 °C overnight. After washing, the diluted secondary biotinylated universal antibody (antiIgG; 1:200) was added, incubated at room temperature for 5 min and a conjugated-streptavidin peroxidase complex was added. The sections were stained with peroxidase substrate solution and then counter-stained with hematoxylin. For immunohistochemistry analysis, the mounting media was added and the sections were embedded in the microscope slides. COX-2 expression was detected if the cytoplasm was stained brown, thus, the cells were considered positive. The level of COX-2 expression was determined based on the number of positive cells according to the previous methods [19,20]. A light microscope with a 1000× magnification was used to calculate the number of cells at five different fields.

#### 2.3.4. Thioglycollate-Induced Leukocytes Migration

This method was performed according to the previous studies [21,22]. HIFPL was tested in the dose of 40, 80 and 160 mg/kg whereas indomethacin was tested at 5 mg/kg. Both HIFPL and indomethacin were dissolved in DMSO to obtain the desired concentrations, and then orally administered (0.2 mL) 30 min prior to the induction of leukocytes migration with 0.5 mL of sterile thioglycollate 4% (intra peritoneal). After 4.5 h, the animals were sacrificed and the peritoneal lavage was collected and further spun in a centrifuge (Sartorius Centrisart G-16C; Goettingen, Germany) at 1200 rpm for 10 min to obtain the cells pellet. The cells pellet was suspended in PBS for total leukocytes counting and cytokines level analysis. The number of cells was counted using a haemocytometer (Assistent; Sandheim, Germany) after staining with methylene blue.

#### 2.3.5. Analysis of Chemokines Level

The amount of chemokines, Interleukine-8 (IL-8) and Monocyte chemoattractant protein-1 (MCP-1) produced in the peritoneum lavage supernatant was quantified using ELISA kit (Cusabio CSB-E07430m and CSB-E07274m, respectively; Cusabio, College Park, MD, USA) according to the protocol from the manufacturers. The activity of HIFPL in reducing the chemokines level was compared to the solvent (DMSO) treatment and presented as percent inhibition.

### 2.4. Cyclooxygenase Enzymatic Assays

A COX Inhibitor Screening Assay kit (Caymann 760111; Cayman Chemical, Ann Arbor, MI, USA) was used to determine the in vitro activity of the HIFPL to inhibit cyclooxygenase enzymes. HIFPL (17.6 mg) was dissolved in 5 mL DMSO to give a stock solution of 3.52 mg/mL. Serial concentrations of HIFPL were prepared by dilution from the stock solution to give final concentrations of 10, 20 and 40 µg/mL in a 96-well plate. Indomethacin stock solution (8.8 mg/10 mL) was prepared in DMSO and tested at final concentrations of 5, 10 and 20 µg/mL. The experiment protocol was done according to the manufacturer’s instructions. This kit was based on colorimetric measurement that included the peroxidase component of cyclooxygenase. The activity of peroxidase is determined by quantification of oxidized tetramethyl-*p*-phenylenediamine (TMPD) generated in the enzymatic reaction at 590 nm in an ELISA reader (Pioway RT-2100C; Nanjing, China) [23]. The percent inhibition was calculated by comparing the inhibitory activity of the test sample-treated group to the inhibitory activity of the solvent-treated group.

### 2.5. Statistical Analysis

The results were presented as a mean ± standard error of mean (SEM) and the statistical analysis was performed to determine the significance of the effects. The data was analyzed using one-way analysis of variance (ANOVA) followed by a post hoc test (Dunnet).

## 3. Results

In this study, we investigated the anti-inflammatory effects of HIFPL using two acute experimental models of inflammation in mice. First, we performed a carrageenan-induced paw edema which represents a common method for an acute anti-inflammatory evaluation. The second in vivo experiment model utilized mice induced by thioglycollate to evaluate the effect of HIFPL on the leukocytes’ migration and chemokines level.

### 3.1. Anti-Inflammatory Activity in Carrageenan-Induced Paw Edema

**Figure 1:**
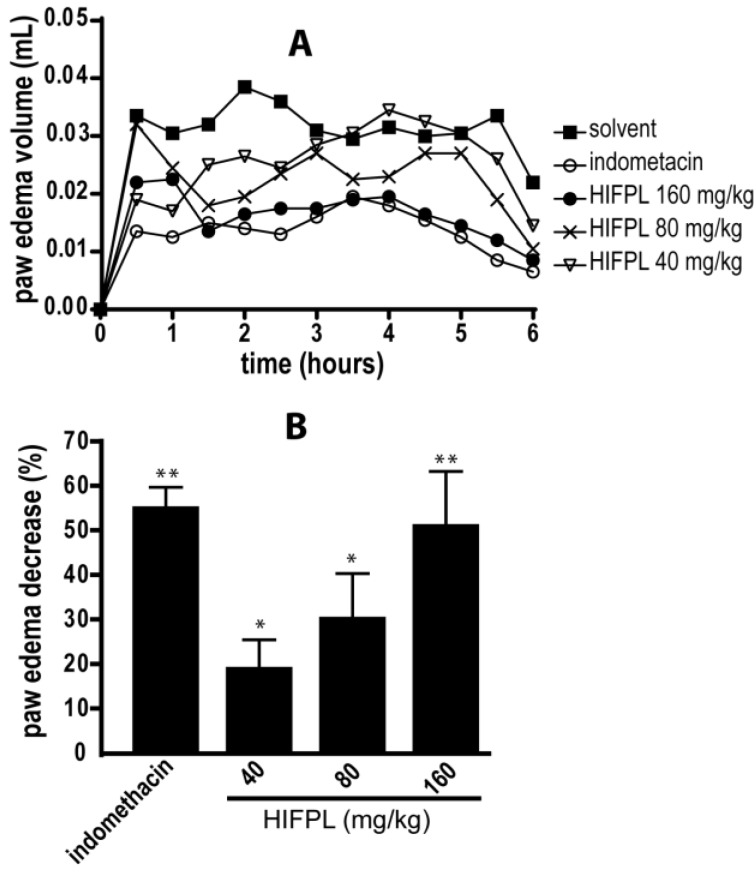
Anti-inflammatory activity of HIFPL on carrageenan-induced paw edema in mice. HIFPL was tested at 40, 80 and 160 mg/kg; indomethacin (5 mg/kg) was used as a reference drug. HIFPL, indomethacin or solvent was given orally 30 min prior to the injection of carrageenan injection (sub plantar). (**A**) Paw edemas were measured every 30 min for 6 h; (**B**) The anti-inflammatory activity of HIFPL was based on the area under the curve (AUC) of each group after 6 h compared to the solvent-treated group. The values are relative AUC ± standard errors. * *p* < 0.05; ** *p* < 0.01 (ANOVA/Dunnett), compared to the solvent-treated group.

HIFPL and indomethacin (positive control) were given orally 30 min prior to carrageenan stimulation. HIFPL was able to reduce the edema volume compared to that of the solvent-treated group (Figure 1) in a dose-dependent manner. This result indicates that HIFPL exerts anti-inflammatory activity in carrageenan-induced paw edema in mice (IC_50_: 21.76 mg/kg). As expected, indomethacin, the reference drug, demonstrated a potent anti-inflammatory activity indicating the sensitivity of this animal experimental model to detect the activity of anti-inflammatory agents.

### 3.2. Analysis of COX-2 Expression

**Figure 2:**
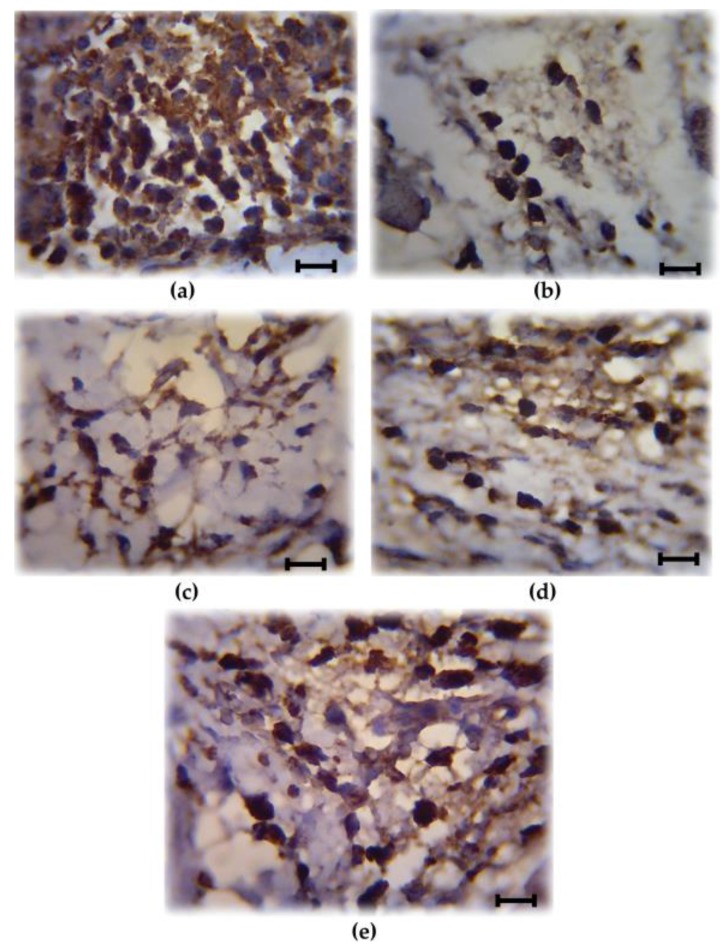
Representative photos showing the cyclooxygenase (COX)-2 expressing cells (stained dark-brown) of the paw edema. (**a**) Solvent-treated group; (**b**) Indomethacin-treated group; (**c**–**e**) HIFPL-treated group at 160, 80 and 40 mg/kg (per oral), respectively. The soft plantar region sections of the hind paw were cut and fixed, COX-2 expression was detected using immonohistochemisty. The number of COX-2 expressing cells were counted in a light microscope with a 1000× magnification at five different fields. Scale bar: 10 μm.

**Figure 3:**
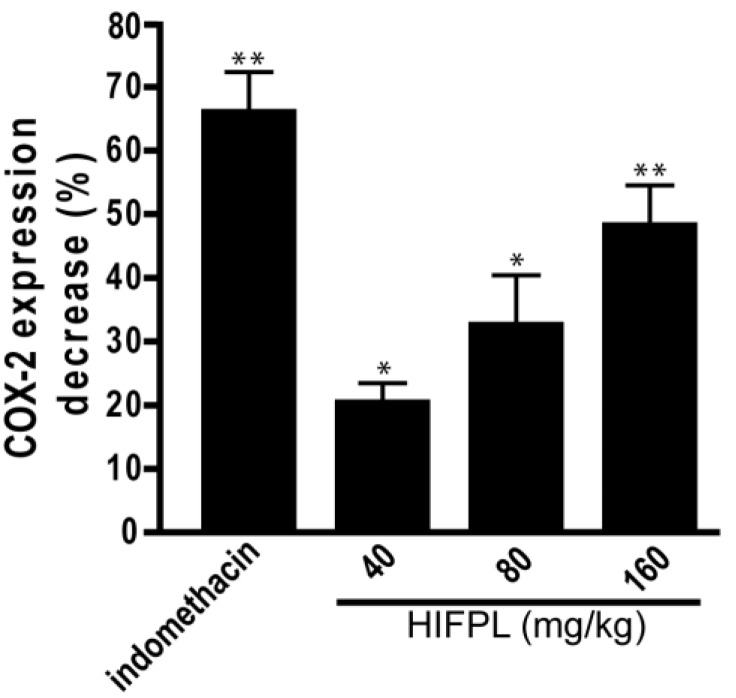
The effect of HIFPL on COX-2 expression in carrageenan-induced paw edema. Mice paws were cut and fixed in buffered formalin and COX-2 expression was quantified. HIFPL was tested at 40, 80 or 160 mg/kg (per oral), and indomethacin (5 mg/kg, per oral) was used as a reference drug. The values are mean ± standard errors. * *p* < 0.05; ** *p* < 0.01 (ANOVA/Dunnett), compared to the solvent-treated group (set as 100% of COX-2 expression).

COX-2 is involved in the production of proinflammatory mediators and involved in the development of paw edema. In order to assess whether the reduction of the paw edema upon HIFPL treatment is due to the inhibition of COX-2, we measured the expression level of COX-2 in the paw edema. The expression of COX-2 was assessed using immunohistochemistry from soft plantar region sections of the hind paw. The COX-2 expression was considered positive if the cytoplasm was stained brown. Figure 2 shows the comparison of COX-2 expression cells (stained dark-brown) in the mice treated with HIFPL, solvent or indomethacin. These photos clearly showed that COX-2 was expressed in the paw edema and the treatment with HIFPL or indomethacin was able to reduce the COX-2 expression. The amount of COX-2 expressing cells was quantified and the result was presented in Figure 3. This figure indicates that pretreatment with HIFPL inhibited COX-2 expression in a dose-dependent manner, suggesting that HIFPL exerts anti-inflammatory activity, partly by inhibiting the expression of COX-2. Consistent with the reduction of paw edema, indomethacin exerts a potent reduction of COX-2 expression.

### 3.3. Cyclooxygenases Inhibition Enzymatic Assays

**Figure 4:**
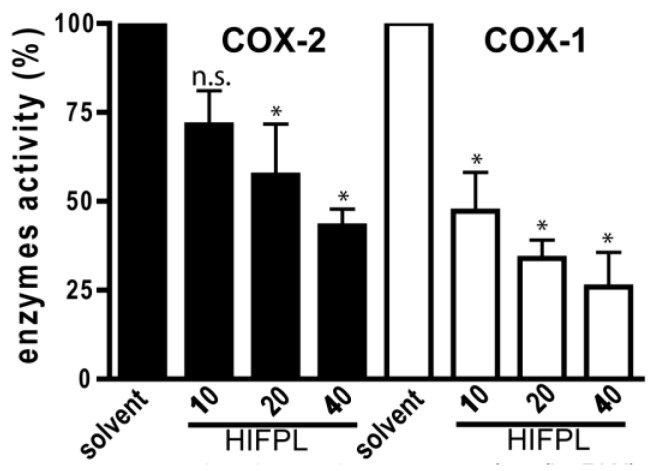
HIFPL inhibited COXs activity in in vitro enzymatic assays. HIFPL was dissolved in DMSO and prepared in three different concentrations (10, 20 and 40 µg/mL). The values are means ± standard errors, * *p* < 0.05; n.s.: not significant (ANOVA/Dunnett), compared to the solvent-treated group.

Not only was the expression of COX-2 investigated, but we also evaluated the effect of HIFPL on COX-2 activity. To determine the selectivity, the effect on the COX-1 was also evaluated. To investigate whether HIFPL also affected the activity of COX-2, in vitro enzymatic assays were performed. We found that HIFPL not only reduced COX-2 expression, but also inhibited the activity of COX-2 in a concentration-dependent manner (Figure 4). To determine the selectivity, HIFPL was also tested for the COX-1 inhibition assay. Our study indicated that HIFPL acted as an unselective COX-2 inhibitor with a higher affinity against COX-1 (IC_50_: 8.13 µg/mL) compared to COX-2 (IC_50_: 35.27 µg/mL).

### 3.4. Leukocytes Migration Assay

**Figure 5:**
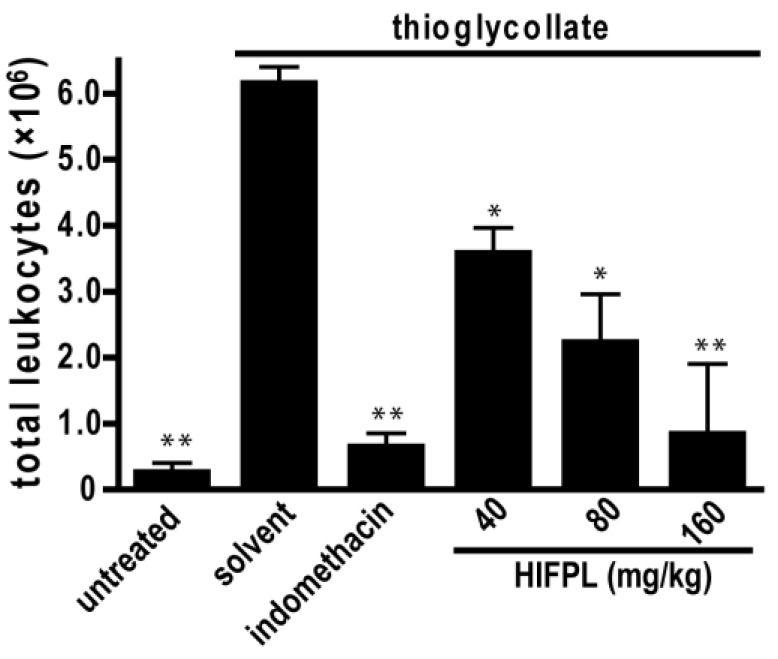
HIFPL inhibited the migration of leukocytes induced by thioglycollate (five mice per group). HIFPL was tested at 40, 80 and 160 mg/kg (per oral) and indomethacin (5 mg/kg, per oral) was used as a reference drug. The values are mean ± standard errors. * *p* < 0.05; ** *p* < 0.01 (ANOVA/Dunnett), compared to the solvent-treated group.

Leukocytes migration plays a crucial role in inflammation. To assess the effect of HIFPL on the early stage of inflammation, we evaluated the effectivity of HIFPL to inhibit leukocytes migration in mice induced by thioglycollate. In this experiment, thioglycollate was able to stimulate the massive migration of leukocytes from the blood circulation to the site of injury located in the peritoneum (Figure 5). The treatment with indomethacin potently reduced leukocytes migration, indicating that this bioassay represents a sensitive method to assess the anti-inflammatory activity of test samples. Consistent with the anti-inflammatory activity in the previous in vivo experiment employing the carrageenan-induced paw edema, HIFPL inhibited leukocytes migration (IC_50_: 61.74 mg/kg) induced by thioglycollate in a concentration-dependent manner. A higher concentration of HIFPL was required to completely inhibit the leukocytes migration to a level similar to the untreated mice.

### 3.5. Analysis of Chemokines Level

**Figure 6:**
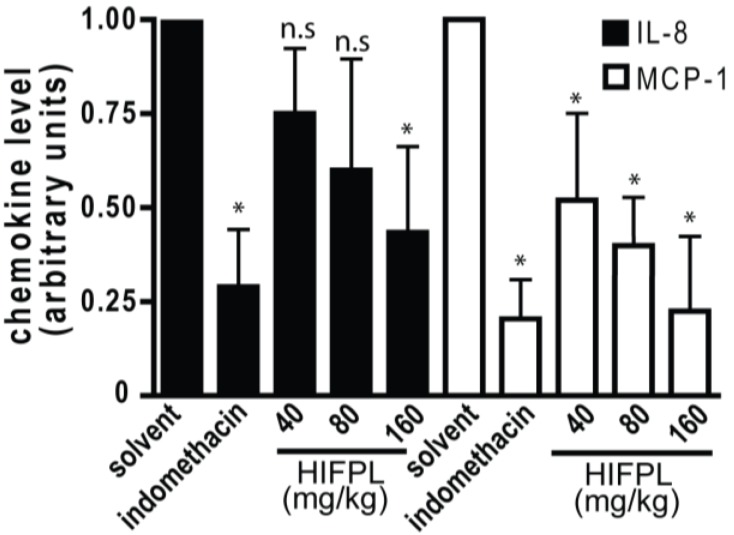
Effect of HIFPL on MCP-1 and IL-8 levels in carrageenan-induced paw edema in mice. HIFPL was tested at 40, 80 and 160 mg/kg (oral administration) and indomethacin (5 mg/kg, oral administration) was used as a reference drug. The values are mean ± standard errors. * *p* < 0.05; n.s.: not significant (ANOVA/Dunnett), compared to the solvent-treated group.

In the inflamed tissues, chemotactic cytokines or chemokines are required for the trafficking of leukocytes. The migration of mice leukocytes induced by thioglycollate was driven by the presence of chemokines such as MCP-1 and IL-8. Thus, we investigated the level of MCP-1 and IL-8 in the mice peritoneum cavity induced by thioglicollate. To evaluate the effect of the tested extract on the chemokine expression, we measured the level of MCP-1 and IL-8 in the HIFPL-treated compared with the solvent-treated mice. Figure 6 demonstrates that the levels of MCP-1 and IL-8 were increased upon thioglycollate induction and the treatment with indomethacin significantly reduced the level of the chemokines. This indicated that these two chemokines were involved in the leukocytes migration process. In line with the ability of HIFPL to inhibit leukocytes migration, the levels of MCP-1 and IL-8 were also decreased upon HIFPL treatment. This suggests that HIFPL inhibited the thioglycollate-induced leukocytes migration in mice peritoneum, at least partly through lowering the level of the chemokines MCP-1 and IL-8.

## 4. Discussion

*Plantago lanceolata* has been widely used worldwide for decades in the preparation of herbal medicine to treat various disorders including inflammatory diseases. Even though the dried leaves were traditionally prepared as a decoct dosage form, previous studies indicated that the dichloromethane extract of the leaves demonstrated a potent inhibition against the main transcription factor in inflammatory events, NF-κB, more potent that the polar extract. To enrich the secondary metabolite components which might be responsible for the activity, the chlorophyll content was removed from the dichloromethane extract of *P. lanceolata* leaves by partition using *n*-hexane to yield a *n*-hexane-insoluble fraction of *P. lanceolata* (HIFPL; Thin layer chromatography profile is shown in Supplementary materials Figure S1). In this study, we investigated the anti-inflammatory activities of HIFPL in mice models to strengthen the scientific evidence regarding the anti-inflammatory activity of this plant. We performed two acute experimental models of inflammation. In the first experiment, we employed a carrageenan-induced paw edema as a common simple method to evaluate the acute anti-inflammatory activity. In this model, the effect of HIFPL on the expression of the enzyme responsible for generating inflammatory mediators, COX-2, was also evaluated. The second experiment model employed the thioglycollate-induced leukocytes migration to confirm the anti-inflammatory efficacy and to get insight into the effect on the proinflammatory chemokines.

We found that HIFPL demonstrates a significant anti-inflammatory effect by reducing paw edema volume in a dose-dependent manner after 6 h of observation, confirming its efficacy as an anti-inflammatory remedy. As edema is one of the common features of an acute inflammatory process [24], the ability of HIFPL to reduce paw edema volume suggested that it has an acute anti-inflammatory activity in mice. However, the activity of HIFPL is still low compared to the reference drug, indomethacin. Further separation of the fraction might be required to obtain a single active compound with stronger anti-inflammatory activity.

The key enzyme in the inflammatory process is COX-2. This inducible enzyme is responsible for generating inflammatory mediators including prostaglandins and leukotrienes from arachidonic acid. In inflammatory condition, COX-2 is over expressed and contributes to the development of inflammatory events. HIFPL significantly reduced COX-2 expression in a dose-dependent manner. However, a higher dose of HIFPL was required to reduce COX-2 expression to the basal level. As expected, indomethacin, the anti-inflammatory drug targeting COXs, demonstrated higher activity. Not only was the expression of COX-2 investigated, but the effect of HIFPL on COX-1 and -2 activities was also determined. Indeed, HIFPL was able to inhibit both COX-2 and COX-1 activities with a high potency. Nevertheless, it demonstrated an unselective COX-2 inhibitor as it also inhibited COX-1 with a higher potency. Previous studies indicated that not only COX-2, but also COX-1 is involved in the progression of neuroinflammation (Alzeimer’s disease) [25,26]. HIFPL can still be considered as a potential source to be developed as a therapeutic agent to prevent or treat neurodegenerative diseases in which neuroinflammation is the underlying pathological mechanism. Interestingly, *P. lanceolata* also inhibits 12-lipooxygenase activity [27], another key enzyme in inflammation, suggesting that *P. lanceolata* has a considerable anti-inflammatory effect and represents a potential plant source of anti-inflammatory compounds.

One of the key events in the progression of acute inflammatory responses is leukocytes migration [28,29]. Thus, inhibition of leukocytes migration is an important approach in combating inflammation [30]. Interestingly, HIFPL effectively inhibited the migration of leukocytes. Again, this confirmed the effectiveness of HIFPL in acute inflammation in vivo experiment models. However, the method used in this study counted the entire leukocytes migration, not a specific type of leukocytes. Thus, further study to differentiate the subset of migrated leukocytes, especially neutrophil, might be necessary. Among the other leukocytes, neutrophil is a main subset that plays a key role in the development of inflammatory processes as well as in various inflammatory-related disorders [31].

The migration of leukocytes to the site of inflammation induced by pro-inflammatory agents such as thioglycollate was driven by the presence of chemokines including IL-8 and MCP-1. HIFPL inhibited the expression level of MCP-1 and IL-8. This finding suggests that HIFPL exerts anti-inflammatory activity in the thioglycollate-induced leukocytes migration in mice, at least partly by lowering the level of MCP-1 and IL-8. As the binding of MCP-1 and IL-8 to their receptors is a crucial event in inflammation and it also represents a potential therapeutic target [32,33,34], the reduction of MCP-1 and IL-8 levels upon HIFPL treatment makes *P. lanceolata* leaves a potential source of natural compounds with promising anti-inflammatory activity. HIFPL exerted a stronger inhibition against MCP-1 compared to IL-1. Considerable data suggested that MCP-1, by its chemotactic activity, contributes to many inflammatory diseases, such as atherosclerosis [35], nephritis [36], periodontitis [37] and arthritis [38]. Therefore, *P. lanceolata* represents a plausible source of bioactive compounds for therapeutic purposes.

Our findings are in line with previous studies showing that *P. lanceolata* leaves exerted anti-inflammatory activity in various models [39,40,41]. Ursolic acid, a major triterpenoid compound of *P. lanceolata* leaves, was likely to be an active compound responsible for the anti-inflammatory activities [42]. Ursolic acid inhibited the activation of transcription factors involved in the inflammation, such as NF-κB, AP-1 (Activator Protein-1), NF-AT (Nuclear Factor of Activated T cells) and STAT3 (Signal Transducer and Activator of Transcription 3) [42,43]. In addition, it was also known to exert anti-inflammatory activities on adjuvant-induced chronic arthritis and zymosan-induced inflammation in mice [44]. In addition, ursolic acid has also been reported as a selective COX-2 inhibitor [45]. Other compounds that might contribute to the anti-inflammatory activity of HIFPL are oleanolic acid, the structural isomer of ursolic acid [45], and the phenylethanoid compounds, plantamajoside and acteoside [46]. These compounds exhibited anti-inflammatory activities in different models.

## 5. Conclusions

We demonstrated that HIFPL exhibited anti-inflammatory activity in in vivo experimental models. It reduced paw edema volume in the carrageenan-induced mice and inhibited the expression and activity of the proinflammatory enzyme COX-2. However, it acts as an unselective COX-2 inhibitor. Interestingly, HIFPL inhibited the migration of leukocytes by reducing the level of chemokines (MCP-1 and IL-8). Our finding is in line with previous studies and correlates with the traditional use of *P. lanceolata* leaves as an anti-inflammatory remedy; this suggests that this plant could be further developed for the discovery of novel leads or dietary supplements targeting inflammation.

## Supplementary Materials

The following are available online at http://www.mdpi.com/2218-0532/85/1/12/s1, Figure S1: Chromatogram (TLC profile) of *P. lanceolata* extract after chlorophyl removal using *n*-hexane partition.
