# Supplementary material for: n-Hexane Insoluble Fraction of Plantago lanceolata Exerts Anti-Inflammatory Activity in Mice by Inhibiting Cyclooxygenase-2 and Reducing Chemokines Levels
Source: Sci Pharm. 2017 Mar 13;85(1):12. doi: 10.3390/scipharm85010012 (PMC5388149; doi:10.3390/scipharm85010012)
Supplement: Supplementary file 1 [file scipharm-85-00012-s001.pdf]

SUPPLEMENTARY MATERIALS

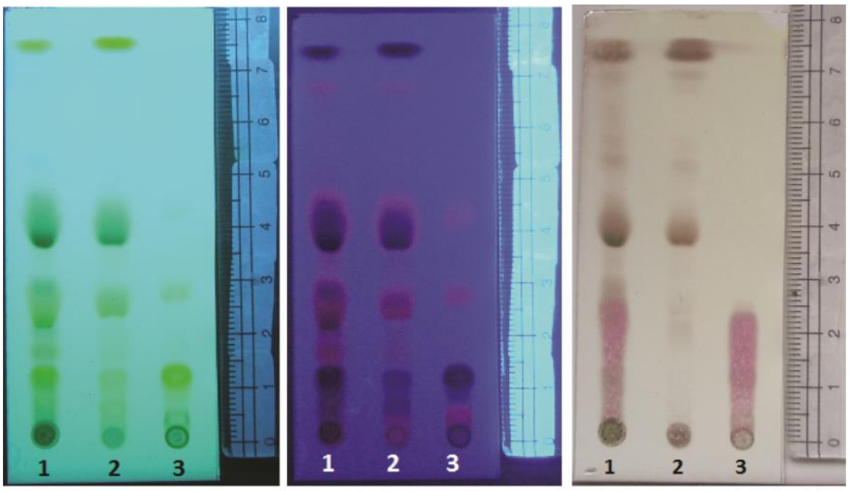

(a) (b) (c)

Figure S1. Chromatogram (TLC profile) of *P. lanceolata* extract after chlorophyl removal using *n*-hexane partition. (1) Dichloromethane extract of *P. lanceolata* leaves; (2) *n*-Hexane-soluble fraction and (3) *n*-Hexane-insoluble fraction (HIFPL) of the *P. lanceolata* leaf extract. Partition of the extract with *n*-hexane resulted in the removal of non-polar constituents, including chlorophylls and other non-polar inert constituents to yield HIFPL. Thin layer chromatography analysis was performed on a silica gel F254 using eluent of *n*-hexane: ethyl acetate (4:1). The TLC spots were detected in UV254 nm **(a)**, UV366 nm **(b)** and after spraying with cerium sulfate followed by heating at 105 °C for 4 minutes **(c)**.
